# Behavioral changes after nicotine challenge are associated with α7 nicotinic acetylcholine receptor-stimulated glutamate release in the rat dorsal striatum

**DOI:** 10.1038/s41598-017-15161-7

**Published:** 2017-11-08

**Authors:** In Soo Ryu, Jieun Kim, Su Yeon Seo, Ju Hwan Yang, Jeong Hwan Oh, Dong Kun Lee, Hyun-Wook Cho, Seong Shoon Yoon, Joung-Wook Seo, Suchan Chang, Hee Young Kim, Insop Shim, Eun Sang Choe

**Affiliations:** 1 Department of Biological Sciences, Pusan National University, 63-2 Busandaehak-ro, Geumjeong-gu, Busan, 46241 Korea; 2 College of Fisheries Sciences, National Institute of Fisheries, 474 Ilgwang-ro, Gijang-gun, Busan, 46041 Korea; 3 Department of Physiology, College of Medicine of Gyeongsang National University, 816-15 Jinju-daero, Jinju, 52727 Korea; 4 Department of Biology, Sunchon National University, 255 Jungang-ro, Sunchon, 57922 Korea; 5 Research Center for Safety Pharmacology, Korea Institute of Toxicology, 141 Gajeong-ro, Yuseong-gu, Daejeon, 34114 Korea; 6 Fundamental Research Division, Korea Institute of Oriental Medicine, 1672 Yuseong-daero, Yuseong-gu, Daejeon, 34054 Korea; 7 College of Korean Medicine, Daegu Haany University, 136 Sincheondong-ro, Suseong-gu, Daegu, 42158 Korea; 8 Department of Science in Korean Medicine, Kyung Hee University, 26 Kyungheedae-ro, Dongdaemun-gu, Seoul, 02447 Korea

## Abstract

Neurochemical alterations associated with behavioral responses induced by re-exposure to nicotine have not been sufficiently characterized in the dorsal striatum. Herein, we report on changes in glutamate concentrations in the rat dorsal striatum associated with behavioral alterations after nicotine challenge. Nicotine challenge (0.4 mg/kg/day, subcutaneous) significantly increased extracellular glutamate concentrations up to the level observed with repeated nicotine administration. This increase occurred in parallel with an increase in behavioral changes in locomotor and rearing activities. In contrast, acute nicotine administration and nicotine withdrawal on days 1 and 6 did not alter glutamate levels or behavioral changes. Blockade of α7 nicotinic acetylcholine receptors (nAChRs) significantly decreased the nicotine challenge-induced increases in extracellular glutamate concentrations and locomotor and rearing activities. These findings suggest that behavioral changes in locomotor and rearing activities after re-exposure to nicotine are closely associated with hyperactivation of the glutamate response by stimulating α7 nAChRs in the rat dorsal striatum.

## Introduction

Tobacco use, such as cigarette smoking, can cause dependence, which is a psychiatric disorder (DSM-V) characterized by compulsive drug intake and various withdrawal symptoms, such as anhedonia, dysphoria, anxiety, irritability, and craving^1–5^. Avoidance of these negative conditions during smoking cessation helps prevent relapse to tobacco use^5–7^. Nicotine, which is the primary psychoactive compound in tobacco, primarily acts on nicotinic acetylcholine receptors (nAChRs; α7 and α4β2 subtypes) in the ventral tegmental area (VTA), substantia nigra pars compacta (SNpc), and prefrontal cortex (PFC)^8–10^. Of these two nAChRs subtypes, the stimulation of α7 nAChRs in the glutamate terminals of the VTA and SNpc potentiates the activity of dopaminergic projections to the dorsal striatum, nucleus accumbens (NAc) and PFC^11,12^. These findings suggest that activation of the dopamine system by stimulating α7 nAChRs has a critical role in drug dependence, including motor function and rewarding properties of drug intake^13,14^.

Glutamate, a key excitatory neurotransmitter in the mammalian brain, has essential roles in the development and maintenance processes of drug dependence. These processes include addictive properties of drugs, such as reinforcement, sensitization, craving, and relapse^15^. Nicotine administration increases the release of glutamate in the VTA, NAc, and PFC via stimulation of nAChRs^16,17^. Increased extracellular glutamate concentrations in the NAc facilitate the intracellular signaling cascades, which are believed to trigger behavioral sensitization^15,18,19^. For instance, repeated and intermittent exposure to nicotine enhances the behavioral response by upregulating the release of glutamate in the NAc and VTA^20^. However, a nicotine-linkage to neurochemical alterations in the rat dorsal striatum has not been sufficiently characterized to date.

Drug, cue, and stress-induced reinstatement of nicotine-seeking behavior have been found to elevate extracellular glutamate levels in the core region of the NAc^21–23^. Blockade of glutamate receptors in the NAc and VTA attenuates the reinstatement of nicotine-seeking behavior^24,25^. These findings suggest that enhancement of glutamatergic neurotransmission in the NAc and VTA appears to be associated with relapse to nicotine use. In addition to the NAc, the dorsal striatum is also a major area for glutamatergic neurotransmission from the somatosensory cortices in the forebrain, which is closely associated with drug-induced locomotion and motivation^26^. Previous microdialysis-based studies have demonstrated that nicotine treatment increases the level of glutamate in the dorsal striatum^27–29^; however, the role of glutamate on nicotine-induced behavioral changes remains to be elucidated. Herein, we demonstrate a novel result, which shows that re-exposure to nicotine increases extracellular glutamate release by stimulating α7 nAChRs in the rat dorsal striatum, which may contribute to alter locomotor and rearing activities.

## Results

### Real-time glutamate biosensing in the dorsal striatum

**Figure 1:**
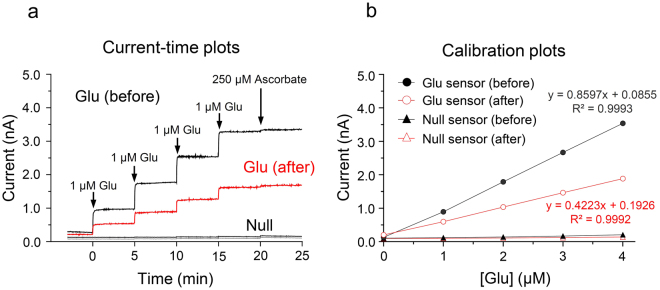
*In vitro* current-time and calibration plots. Current-time plots obtained from successive addition of a glutamate standard solution in PBS at pH 7.4, before and after biosensing (**a**). Calibration plots of current-glutamate concentrations [Glu] detected by the glutamate biosensors and null biosensors *in vitro* (**b**). These changes in the currents of the dorsal striatum were then converted into changes in the concentrations of glutamate based on the sensitivity of each glutamate biosensor adjusted by its calibration plots. Glu, Glutamate; Glu biosensor, _L_-glutamate oxidase-based glutamate biosensor; Null biosensor, _L_-glutamate oxidase-free glutamate biosensor.

The sensitivity of the glutamate biosensors prior to biosensing was 0.813 ± 0.044 nA/μM, while it was approximately two-fold lower (0.352 ± 0.026 nA/μM) after-sensing (Fig. 1a). A single addition of ascorbate did not interfere with glutamate detection *in vitro* (before-sensing: 0.064 ± 0.086 nA/μM; after-sensing: 0.056 ± 0.004 nA/μM). Linear calibration plots were obtained from steady-state currents output at glutamate concentrations in the range of 0–4 μM (Fig. 1b). There were no significant changes in steady-state currents or glutamate concentrations in response to the addition of glutamate standard solutions during *in vitro* calibration of the glutamate null biosensors (before-sensing: 0.024 ± 0.005 nA/μM; after-sensing: 0.011 ± 0.002 nA/μM) (Fig. 1a and b).

### Repeated nicotine administration, but not acute nicotine, significantly increased extracellular glutamate concentrations

**Figure 2:**
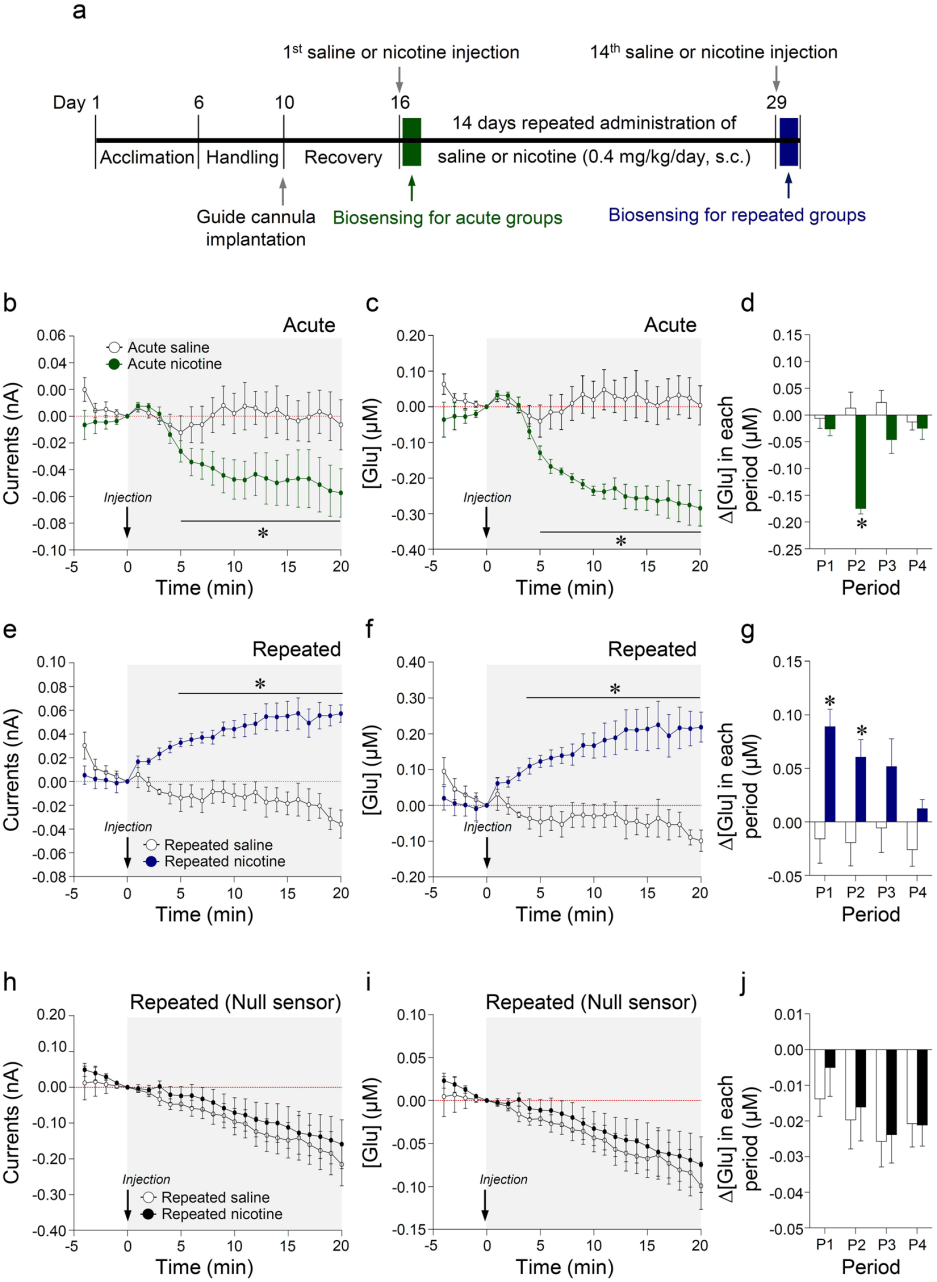
Changes in output currents and glutamate concentrations in the dorsal striatum after acute and repeated administration of nicotine. Timelines for acute and 14 day repeated administration of saline or nicotine (0.4 mg/kg/day) and real-time glutamate biosensing (**a**). Changes in output currents (**b**,**e**,**h**) that are converted to glutamate concentrations (**c**,**f**,**i**) and the rate of change in glutamate concentration (Δ[Glu]) (**d**,**g**,**j**) after acute and repeated administrations of saline or nicotine in the dorsal striatum. *p < 0.05 versus saline control group. P1, 0–5 min; P2, 5–10 min; P3, 10–15 min; P4, 15–20 min; n = 5 per group.

The timelines for real-time biosensing of extracellular glutamate after acute or 14 days of repeated saline or nicotine administration in freely moving rats are illustrated in Fig. 2a. The results demonstrated that acute nicotine administration significantly decreased output currents (Fig. 2b) and glutamate concentrations (Time, F_(24,96)_ = 11.88, p < 0.0001; Treatment, F_(1,4)_ = 16.78, p = 0.0149; Time × Treatment, F_(24,96)_ = 7.955, p < 0.0001) (Fig. 2c) compared to those in the saline control group. To determine the rate of change in glutamate concentrations (Δ[Glu]) after saline or nicotine administration, we analyzed the data from four time periods (P1: 0–5 min, P2: 5–10 min, P3: 10–15 min, P4: 15–20 min). The rate of change in glutamate concentration after acute nicotine administration was significantly decreased at P2 (t_(8)_ = 5.964, p = 0.0003), but not at P1, P3, and P4 (Fig. 2d). In contrast, repeated nicotine administration significantly increased output currents (Fig. 2e) and glutamate concentrations (Time, F_(24,96)_ = 2.599, p = 0.0005; Treatment, F_(1,4)_ = 10.54, p = 0.0315; Time × Treatment, F_(24,96)_ = 12.06, p < 0.0001) (Fig. 2f). The rate of change in glutamate concentration after repeated nicotine administration was significantly increased at P1-P2 (P1, t_(8)_ = 3.736, p = 0.0057; P2, t_(8)_ = 2.920, p = 0.0193), but not at P3-P4 (Fig. 2g).

To determine whether the increase in glutamate concentration occurred as a specific response of glutamate biosensors to extracellular glutamate in the dorsal striatum, we performed glutamate biosensing with glutamate null biosensors after repeated administration of saline or nicotine. The results demonstrated that there were no significant changes in output currents, glutamate concentrations, or rates of change in glutamate concentration in all time periods after repeated nicotine administration (Fig. 2h–j). These data indicate that nicotine-induced current changes in the dorsal striatum, as detected by glutamate biosensors, were a specific response to extracellular glutamate. Thus, following experiments were conducted with glutamate biosensors only.

### Nicotine challenge administration, but not the 1^st^ or 6^th^ day of nicotine withdrawal, significantly increased extracellular glutamate concentrations

**Figure 3:**
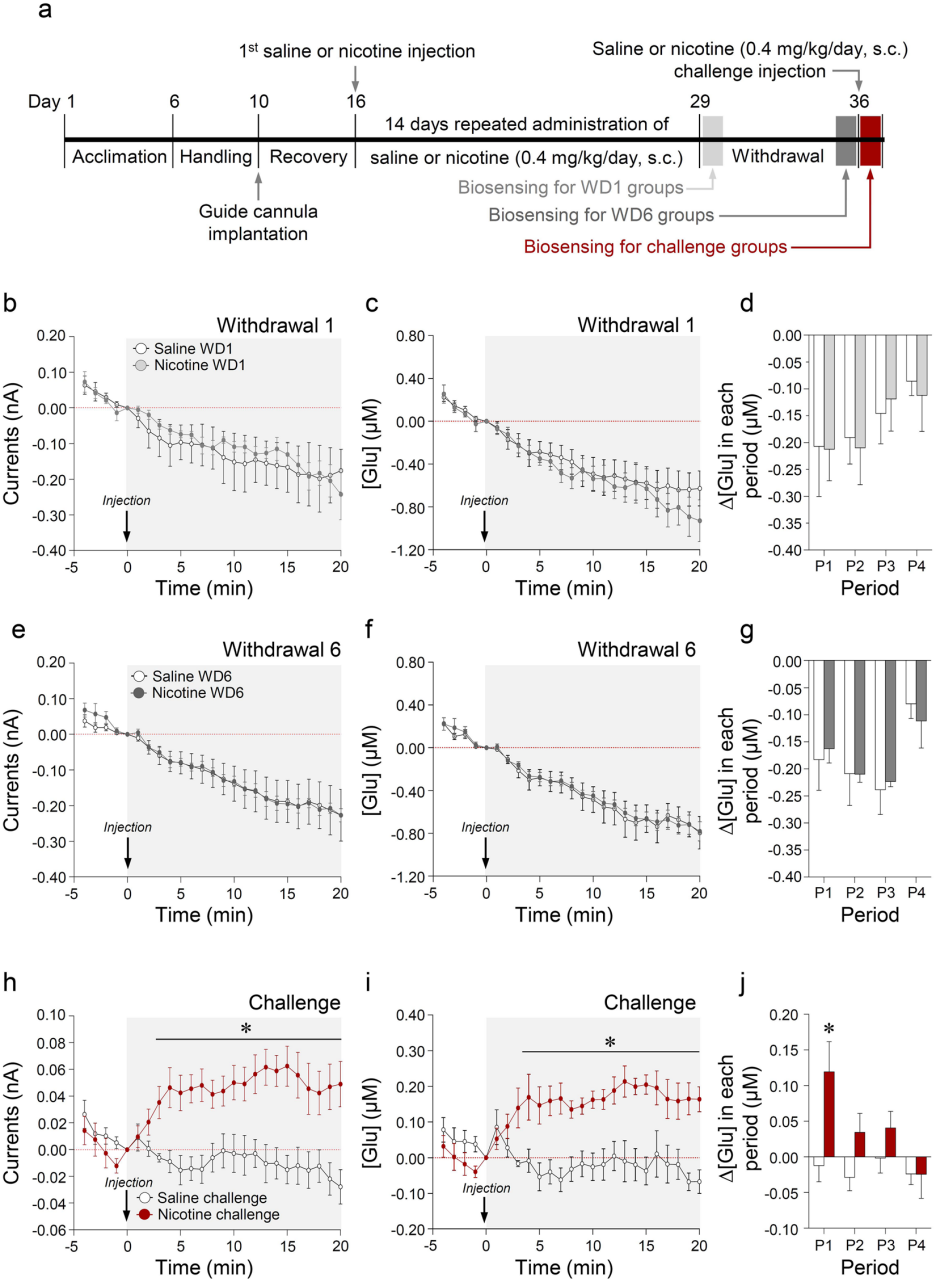
Changes in output currents and glutamate concentrations in the dorsal striatum after withdrawal treatment and challenge administration of nicotine. Timelines for withdrawal or challenge injection of saline or nicotine (0.4 mg/kg/day) and real-time glutamate biosensing (**a**). Changes in output currents (**b**,**e**,**h**) that are converted to glutamate concentrations (**e**,**f**,**i**), and rates of change in glutamate concentration (**d**,**g**,**j**) in the dorsal striatum on the 1^st^ and 6^th^ day of withdrawal and after challenge administration of saline or nicotine. *p < 0.05 versus saline control. P1, 0–5 min; P2, 5–10 min; P3, 10–15 min; P4, 15–20 min; n = 5 per group.

The timelines for the real-time biosensing of extracellular glutamate concentrations after withdrawal or challenge administration of saline or nicotine in freely moving rats are shown in Fig. 3a. The results demonstrated that there were no changes in output currents (Fig. 3b and e) and glutamate concentrations in the 1^st^ and 6^th^ day of nicotine withdrawal (Fig. 3c and f). Moreover, there were no changes in the rate of change of glutamate concentration at all time periods in the 1^st^ and 6^th^ day of nicotine withdrawal (Fig. 3d and g). Similar to the results from repeated nicotine exposure (Fig. 2e and f), nicotine challenge administration significantly increased the output currents (Fig. 3h) and glutamate concentrations (Time, F_(24,96)_ = 2.536, p = 0.0007; Treatment, F_(1,4)_ = 17.87, p = 0.0134; Time × Treatment, F_(24,96)_ = 6.824, p < 0.0001) (Fig. 3i). The rate of change in glutamate concentration after nicotine challenge administration significantly increased at P1 (t_(8)_ = 2.744, p = 0.0253), but not at P2-P4 (Fig. 3j). The absolute values of the rates of change in glutamate concentration in the dorsal striatum at each period throughout the acute, repeated, withdrawal and challenge administrations are listed in Supplementary Table S1.

### Repeated and challenge administrations of nicotine significantly increased locomotor and rearing activities

**Figure 4:**
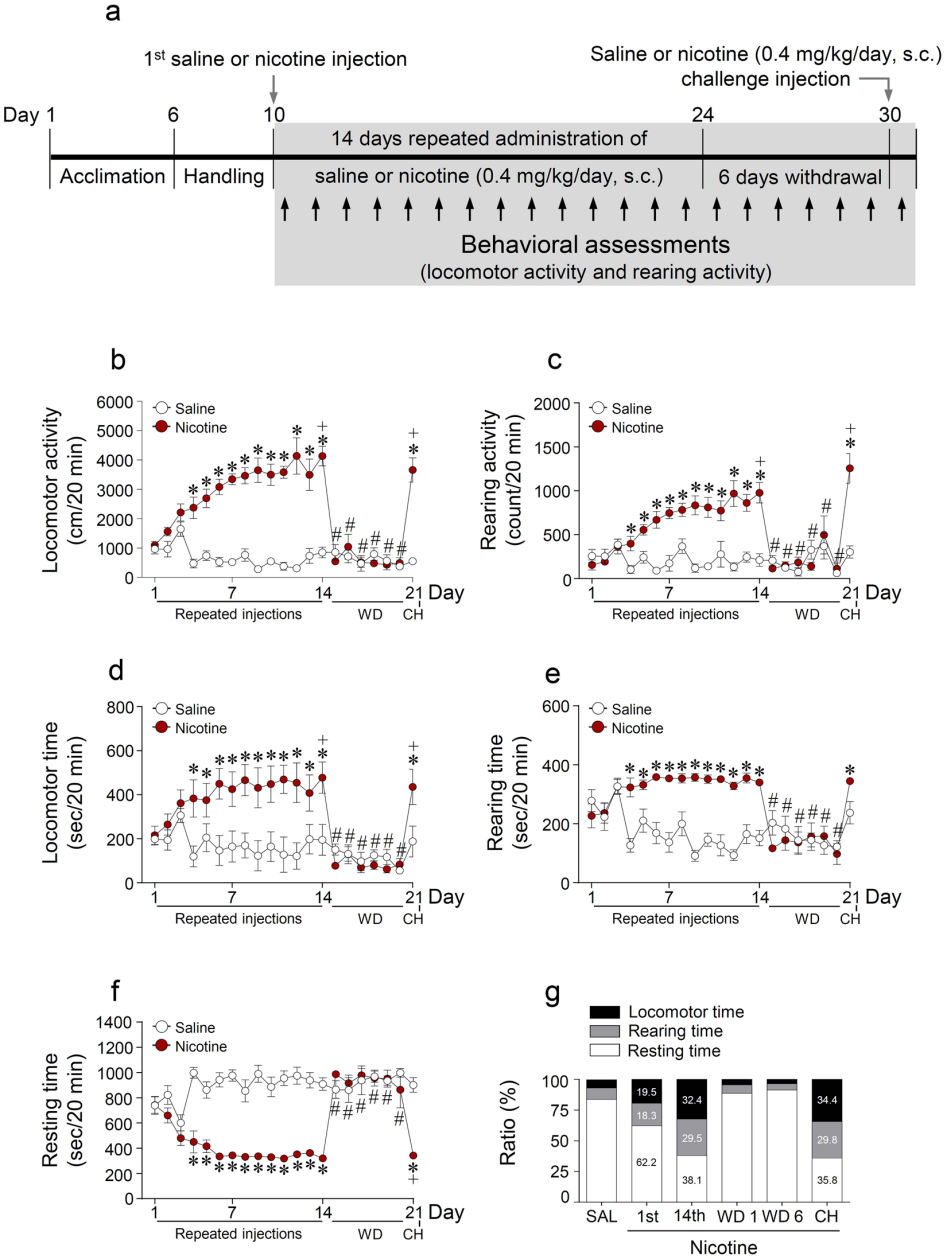
Changes in behavioral activities after acute, repeated, withdrawal, and challenge injection of nicotine. A timeline for behavioral assessments (black arrows) after saline or nicotine (0.4 mg/kg/day) administration (**a**). Changes in the locomotor activity (**b**), rearing activity (**c**), locomotor time (**d**), rearing time (**e**), resting time (**f**), and relative ratios (**g**) for 20 min after every injection throughout all experiments. *p < 0.05 versus saline control group (two-tailed unpaired t test); ^#^p < 0.05, versus 14^th^ days repeated nicotine group; ^+^p < 0.05 versus 4^th^ days repeated nicotine group. n = 6 per group.

Behavioral assessments timelines for locomotor and rearing activities are shown in Fig. 4a. The results showed that repeated nicotine treatment for 14 days and nicotine challenge administrations significantly increased locomotor activity (Time, F_(20,100)_ = 13.93, p < 0.0001; Treatment, F_(1,5)_ = 333.1, p < 0.0001; Time × Treatment, F_(20,100)_ = 20.41, p < 0.0001) (Fig. 4b) and rearing activity (Time, F_(20,100)_ = 8.958, p < 0.0001; Treatment, F_(1,5)_ = 61.05, p = 0.0006; Time × Treatment, F_(20,100)_ = 9.961, p < 0.0001) (Fig. 4c). Repeated nicotine and nicotine challenge administrations also significantly increased locomotor time (Time, F_(20,100)_ = 10.52, p < 0.0001; Treatment, F_(1,5)_ = 7.705, p = 0.0391; Time × Treatment, F_(20,100)_ = 7.104, p < 0.0001) (Fig. 4d) and rearing time (Time, F_(20,100)_ = 6.144, p < 0.0001; Treatment, F_(1,5)_ = 49.00, p = 0.0009; Time × Treatment, F_(20,100)_ = 10.65, p < 0.0001) (Fig. 4e). However, parallel, it significantly decreased resting time (Time, F_(20,100)_ = 11.13, p < 0.0001; Treatment, F_(1,5)_ = 194.3, p < 0.0001; Time × Treatment, F_(20,100)_ = 13.69, p < 0.0001) (Fig. 4f) compared to that in the saline control groups. The relative ratios (locomotor time: rearing time: resting time) at the five different administration phases as follows: (1) saline (6.8: 9.1: 84.1); (2) acute nicotine (19.5: 18.3: 62.2); (3) repeated nicotine (32.4: 29.5: 38.1); (4) 1^st^ day of withdrawal after repeated nicotine (4.6: 6.7: 88.7); (5) 6^th^ day of withdrawal after repeated nicotine (3.7: 5.1: 91.2); and (6) nicotine challenge (34.4: 29.8: 35.8) (Fig. 4g).

**Figure 5:**
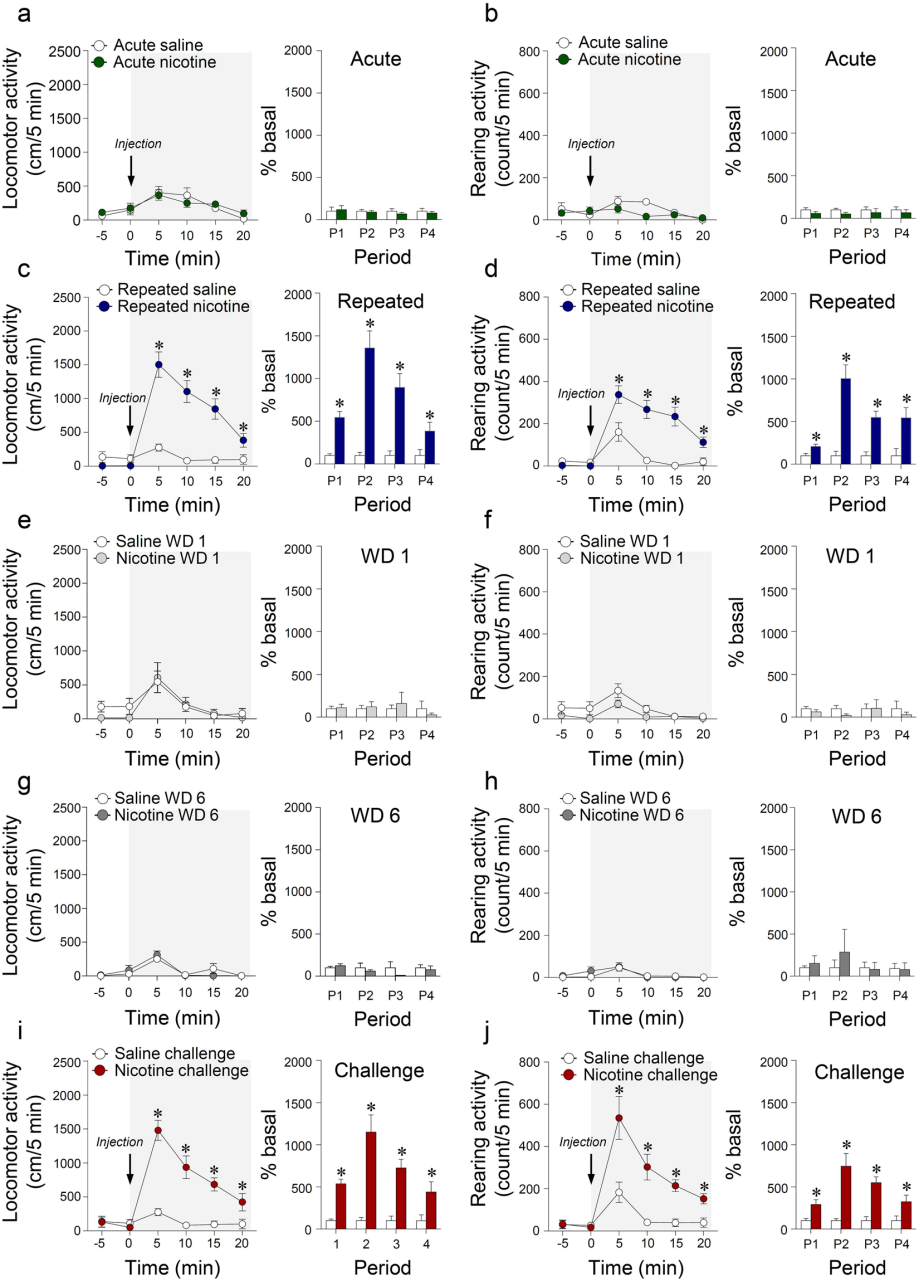
Changes in locomotor and rearing activities after acute, repeated, withdrawal, and challenge administration of nicotine. Changes in locomotor and rearing activities after acute (**a**,**b**), 14 day repeated (**c**,**d**), the 1^st^ day of withdrawal (**e**,**f**), the 6^th^ day of withdrawal (**g**,**h**), and challenge (**i**,**j**) administrations of saline or nicotine (0.4 mg/kg/day). *p < 0.05 versus saline control. P1, 0–5 min; P2, 5–10 min; P3, 10–15 min; P4, 15–20 min; n = 6 per group.

To determine if the glutamate response to nicotine was correlated with the observed behavioral changes, we divided the behavioral change period after saline or nicotine administration into 5 min intervals within each of the five different administration phases. Acute nicotine administration did not alter locomotor and rearing activities (Fig. 5a and b). Similar to the results for the rate of change in glutamate levels, repeated nicotine administration significantly increased locomotor activity (Time, F_(5,25)_ = 31.54, p < 0.0001; Treatment, F_(1,5)_ = 167.2, p < 0.0001; Time × Treatment, F_(5,25)_ = 21.74, p < 0.001) (P1, t_(10)_ = 6.314, p < 0.0001; P2, t_(10)_ = 6.222, p < 0.0001; P3, t_(10)_ = 4.728, p = 0.0008; P4, t_(10)_ = 2.310, p = 0.0435) (Fig. 5c) and rearing activity (Time, F_(5,25)_ = 27.52, p < 0.0001; Treatment, F_(1,5)_ = 60.56, p = 0.0006; Time × Treatment, F_(5,25)_ = 20.48, p < 0.0001) (P1, t_(10)_ = 2.910, p = 0.0156; P2, t_(10)_ = 5.350, p = 0.0003; P3, t_(10)_ = 5.309, p = 0.0003; P4, t_(10)_ = 3.035, p = 0.0126) (Fig. 5d) at each period compared to the levels in the repeated saline group. In contrast, withdrawal treatments did not significantly change locomotor and rearing activities (Fig. 5e–h). Similar to the results from repeated nicotine exposure for 14 days, nicotine challenge administration also significantly increased locomotor activity (Time, F_(5,25)_ = 31.51, p < 0.0001; Treatment, F_(1,5)_ = 76.03, p = 0.0003; Time × Treatment, F_(5,25)_ = 20.09, p < 0.0001) (P1, t_(10)_ = 7.705, p < 0.0001; P2, t_(10)_ = 5.037, p = 0.0005; P3, t_(10)_ = 5.372, p = 0.0003; P4, t_(10)_ = 2.425, p = 0.0357) (Fig. 5i) and rearing activity (Time, F_(5,25)_ = 15.80, p < 0.0001; Treatment, F_(1,5)_ = 47.28, p = 0.0010; Time × Treatment, F_(5,25)_ = 11.09, p < 0.0001) (P1, t_(10)_ = 3.120, p = 0.0109; P2, t_(10)_ = 4.247, p = 0.0017; P3, t_(10)_ = 5.309, p = 0.0003; P4, t_(10)_ = 2.339, p = 0.0414) (Fig. 5j) at all time periods. The real values of the changes in locomotor and rearing activities at each period throughout the acute, repeated, withdrawal, and challenge administrations are listed in Supplementary Table S2.

Throughout the experiments, post-operated processes, such as implantation of glutamate probe, attachment of Rat Hat Bottom/potentiostat, and etc. did not affect behavior (Supplementary Fig. S1).

### Changes in extracellular glutamate concentration in the dorsal striatum were correlated with locomotor and rearing activities after repeated and challenge administrations of nicotine

**Figure 6:**
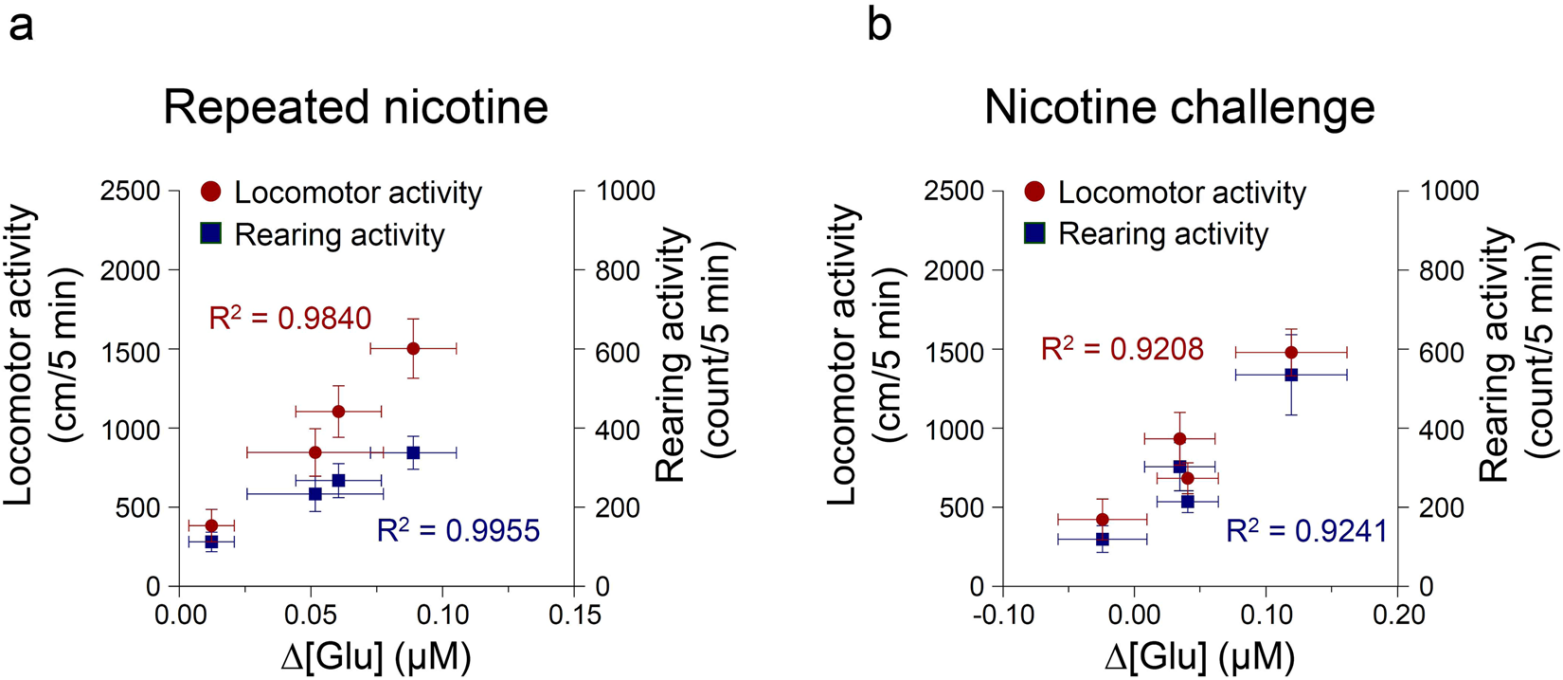
Correlation coefficients for the relationships between the rate of change in glutamate concentration of the dorsal striatum and behavioral changes. Repeated nicotine administration for 14 days and the nicotine challenge-induced increase in the rate of change in glutamate concentration in the dorsal striatum have high correlation coefficients with repeated or nicotine challenge-induced increases in locomotor and rearing activities (p < 0.05) (**a**,**b**). Left y-axis and right y-axis indicate real values for the locomotor and rearing activities, respectively; n = 6 per group.

The results demonstrated that there were high correlation coefficients between changes in extracellular glutamate concentration and changes in locomotor (Pearson correlation test, p = 0.0081, R^2^ = 0.9840) and rearing (p = 0.0023, R^2^ = 0.9955) activities (Fig. 6a). Similarly, changes in glutamate concentration after nicotine challenge administration showed high correlation coefficients with changes in locomotor (p = 0.0404, R^2^ = 0.9208) and rearing (p = 0.0387, R^2^ = 0.9241) activities (Fig. 6b).

### Repeated cotinine administration did not alter locomotor and rearing activities

Cotinine, an alkaloidal tobacco constituent, is a predominant metabolite of nicotine^30^. It has a chemical structure closely related to nicotine and has psychoactive properties through stimulation of nAChRs^31–33^. This part of the study was conducted to determine if cotinine, a nicotine metabolite, administered in periphery after repeated nicotine exposure contributes to behavioral changes in locomotor and rearing activities. The timelines for behavioral assessments after repeated saline or cotinine injections are shown in Supplementary Fig. S2a. The results demonstrated that, compared to the repeated saline group, there were no changes in locomotor and rearing activities in the cotinine-treated group at each tested period (Supplementary Fig. S2b–e). The real values of changes in locomotor and rearing activities at each period after repeated cotinine administration are listed in Supplementary Table S3.

### Blockade of α7 nAChRs significantly decreased the nicotine challenge-induced increase in the release of extracellular glutamate in the dorsal striatum

**Figure 7:**
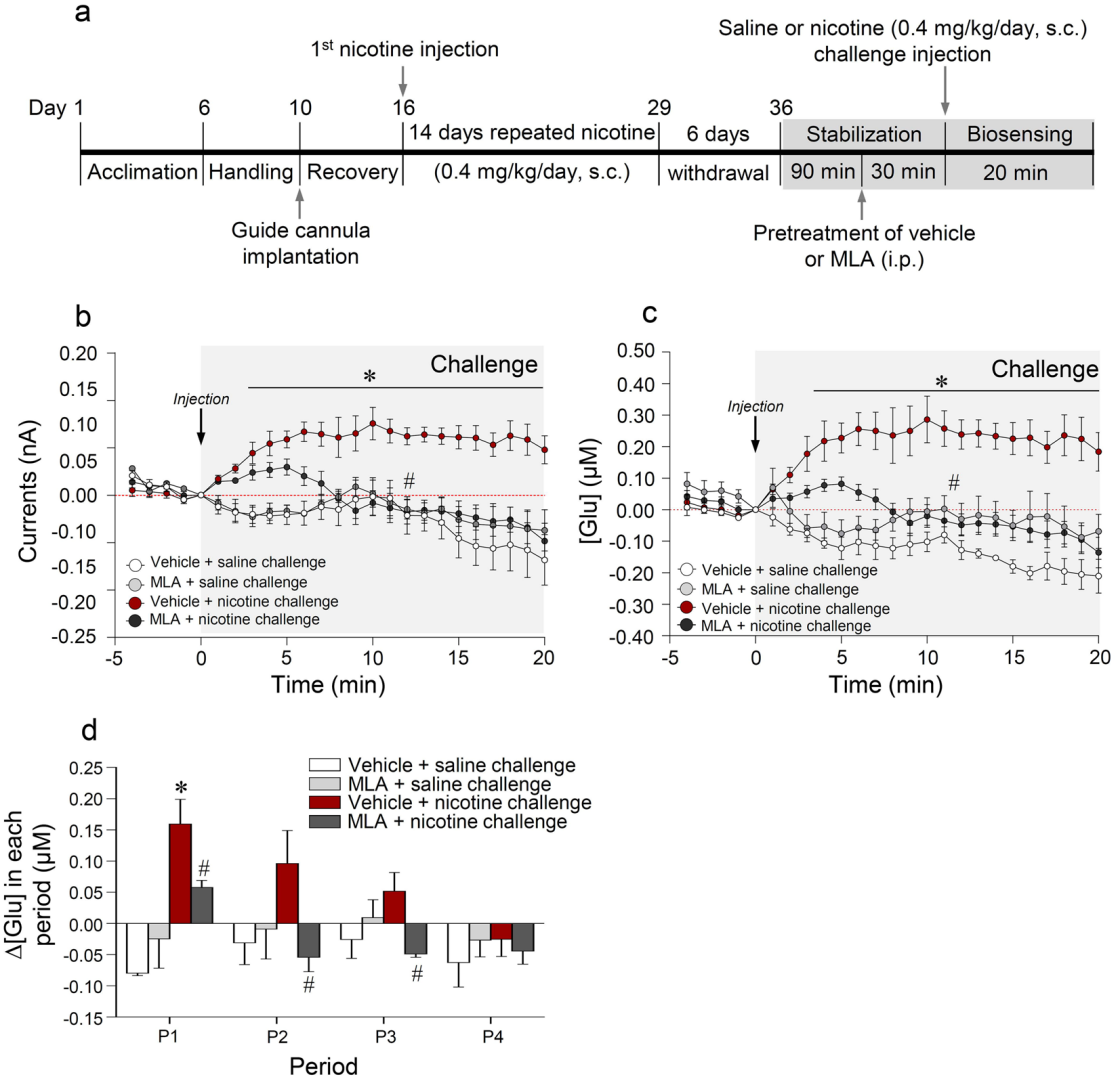
Timelines for real-time glutamate biosensing in the dorsal striatum after challenge administration of saline or nicotine (0.4 mg/kg/day) followed by systemic administration of MLA (10 mg/kg/day) (**a**). The MLA effect on the nicotine challenge-induced increase in output currents (**b**), converted glutamate concentrations (**c**), and rates of change in glutamate concentration at P1-P4 (**d**). *p < 0.05 versus 14 days repeated nicotine + 6^th^ day of withdrawal + vehicle + saline challenge control group; ^#^p < 0.05, versus 14 days repeated nicotine + 6^th^ day of withdrawal + vehicle + nicotine challenge group. P1, 0–5 min; P2, 5–10 min; P3, 10–15 min; P4, 15–20 min; n = 6 per group.

The timelines for real-time biosensing of extracellular glutamate release in the dorsal striatum after saline or nicotine challenge administration, followed by nicotine abstinence, are shown in Fig. 7a. The results demonstrated that treatment with the potent α7 nAChRs antagonist, methyllycaconitine citrate (MLA), prior to nicotine challenge administration significantly decreased the nicotine challenge-induced increases in output currents (Fig. 7b) and glutamate concentrations (Time, F_(24,96)_ = 4.345, p < 0.0001; Treatment, F_(1,4)_ = 38.88, p = 0.0034; Time × Treatment, F_(24,96)_ = 8.345, p < 0.0001) (Fig. 7c) compared to those resulting from nicotine challenge administration followed by vehicle pretreatment. However, there were no changes in output currents and glutamate concentrations after challenge saline administration followed by vehicle, or by MLA pretreatment after nicotine withdrawal (Fig. 7b and c). However, treatment with MLA prior to nicotine challenge administration significantly decreased the nicotine challenge-induced increase in the rate of change in glutamate concentration at P1-P3 (P1, t_(8)_ = 2.471, p = 0.0386; P2, t_(8)_ = 2.599, P = 0.0317; P3, t_(8)_ = 3.302, P = 0.0108), but not at P4 (Fig. 7d). The absolute values for the rates of change in glutamate concentration in the dorsal striatum at each period are listed in Supplementary Table S4.

### Blockade of α7 nAChRs significantly decreased the nicotine challenge-induced increase in locomotor and rearing activities

**Figure 8:**
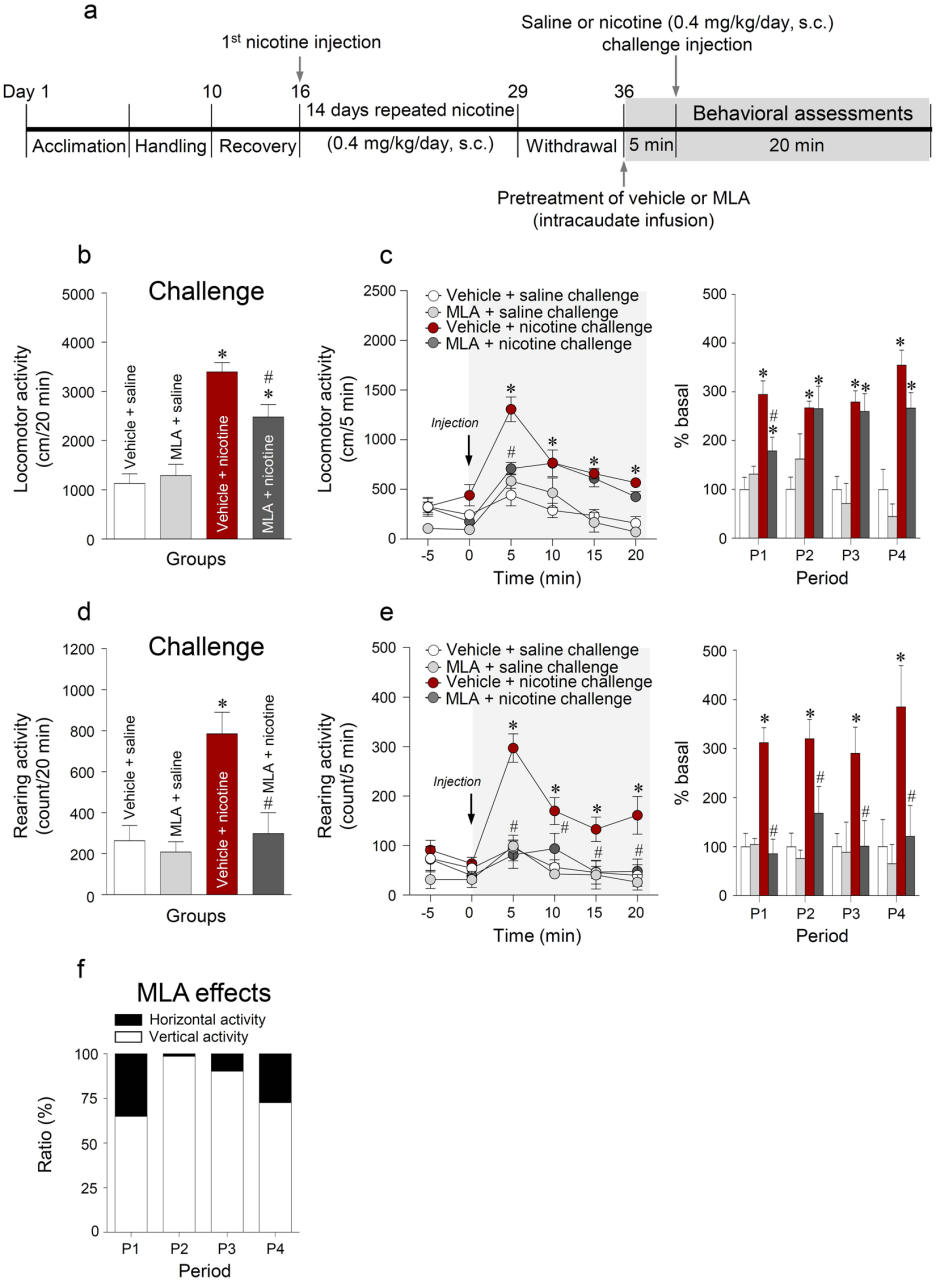
Timelines for behavioral assessments following saline or nicotine (0.4 mg/kg/day) challenge administration followed by intracaudate infusion of MLA (10 μg/μL/side) (**a**). MLA effects on the nicotine challenge-induced changes in locomotor activity (**b**,**c**), rearing activity (**d**,**e**), and relative ratios (**f**) for 20 min after challenge administration of saline or nicotine. *p < 0.05 versus 14 days repeated nicotine + 6^th^ day of withdrawal + vehicle + saline challenge control group; ^#^p < 0.05 versus 14 days repeated nicotine + 6^th^ day of withdrawal + vehicle + nicotine challenge group. P1, 0–5 min; P2, 5–10 min; P3, 10–15 min; P4, 15–20 min; n = 6 per group.

The timelines for vehicle or MLA pretreatment and behavioral assessments are shown in Fig. 8a. The results demonstrated that MLA pretreatment significantly decreased the nicotine challenge-induced increase in locomotor (F_(3,20)_ = 23.28, p < 0.0001) (Fig. 8b) and rearing (F_(3,20)_ = 9.589, p = 0.0004) activities (Fig. 8d). The results of the analysis of post-administration 5 min intervals showed that MLA pretreatment decreased the locomotor activity (Time, F_(5,25)_ = 25.98, p < 0.0001; Treatment, F_(1,5)_ = 6.737, p = 0.0485; Time × Treatment, F_(5,25)_ = 4.099, p = 0.0074) at P1 (t_(10)_ = 2.921, p = 0.0153), but not at P2-P4 (Fig. 8c), and decreased rearing activity (Time, F_(5,25)_ = 10.93, p < 0.0001; Treatment, F_(1,5)_ = 14.75, p = 0.0121; Time × Treatment, F_(5,25)_ = 4.596, p = 0.0041) at P1-P4 (P1, t_(10)_ = 5.386, p = 0.0003; P2, t_(10)_ = 2.258, p = 0.0475; P3, t_(10)_ = 2.539, p = 0.0294; P4, t_(10)_ = 2.515, p = 0.0306) (Fig. 8e) when compared to the results from nicotine challenge followed by vehicle pretreatment. The relative ratios (locomotor activity: rearing activity) of MLA effects on the reduction of locomotor and rearing activities after nicotine challenge administration are as follows: (1) P1 (35.1: 64.9); (2) P2 (1.5: 98.5); (3) P3 (9.8: 90.2); and (4) P4 (27.4: 72.6) (Fig. 8f). These results demonstrated that the stimulation of α7 nAChRs in the dorsal striatum regulates vertical (rearing) activity to a greater extent than horizontal (locomotor) activity following nicotine challenge administration. The real values of the changes in locomotor and rearing activities at each period are listed in Supplementary Table S5.

## Discussion

The dorsal striatum receives nigrostriatal dopaminergic projections from the SNpc, as well as glutamatergic projections from several brain areas, including the somatosensory cortices, amygdala, and hippocampus^34^, indicating an integration of dopaminergic and glutamatergic neurotransmissions in the dorsal striatum. Long-term exposure to nicotine enhances its reinforcing property by stimulating the excitatory α7 nAChRs, which leads to a glutamate release in the VTA, NAc, PFC, and hippocampus^8,35,36^. Microdialysis-based analyses have demonstrated that nicotine administration causes increases in glutamate release in the dorsal striatum, NAc, and VTA^13,27–29,37^. Consistent with these findings, the present data demonstrate that repeated exposure to nicotine increases the extracellular glutamate concentration in the dorsal striatum. These findings suggest that prolonged stimulation of α7 nAChRs after repeated nicotine administration is required for the enhancement of glutamatergic neurotransmission in the dorsal striatum. In contrast to the results of repeated nicotine administration, the present data show that acute exposure to nicotine decreases extracellular glutamate concentrations. The nAChRs containing α4 and β2 subunits, the two most prominent subtypes of nAChRs in the mammalian brain, are densely expressed in the thalamus, cortical region, caudate, and cerebellum^38^. Chronic nicotine treatment increases the affinity of nAChRs with α4 and β2 subunits in the cerebral cortex, caudate putamen, and VTA of mice^39^. The α4β2 nAChRs, which are densely expressed in γ-aminobutyric acid (GABA)ergic neurons, modulate the release of GABA in the VTA of rat^40^. Based on these findings, acute nicotine exposure appears to stimulate GABAergic neurons rather than glutamatergic neurons because of nicotine’s affinity to nAChRs. Acute nicotine exposure may cause hyposensitization of the glutamate response in the dorsal striatum.

Glutamate is a crucial mediator of drug-induced synaptic plasticity, leading to the development and maintenance of drug addiction^15^. Repeated intravenous injections of nicotine result in an elevation of glutamate levels in the NAc and VTA of rat^20^. In addition, nicotine treatment decreases glutamate reuptake via downregulation of glutamate transporter type 3 activity *in vitro* ^41^. The results of the present study demonstrate that 14 days of repeated, but not acute, nicotine administration increases the concentration of extracellular glutamate in the dorsal striatum. Taken together, these findings suggest that enhancement of glutamatergic neurotransmission in the dorsal striatum after repeated nicotine exposure is associated with stimulation of nAChRs.

Nicotine withdrawal reflects adaptive changes in the glutamatergic and cholinergic systems, which appear to enhance craving^12,42^. For instance, downregulation of metabotropic glutamate receptor 2/3 (mGluR2/3) functions during early nicotine withdrawal followed by nicotine self-administration results in impaired negative feedback control of glutamate release in the NAc, which may lead to a hypersensitive glutamate response to nicotine^42^. Desensitization of nAChRs is recovered in receptor functions during nicotine withdrawal in the hippocampus^43^. Herein, it was shown that repeated and challenge administrations of nicotine increased the concentration of glutamate, while nicotine withdrawal produced no such increase. Based on the limited evidence obtained from this study, it can be speculated that adaptive changes in neurochemical systems, including mGluR2/3 and α7 nAChRs, evoke the glutamate response when re-exposure to nicotine, even though behavioral adaptation may not be involved.

Several studies have shown that behavioral changes in response to psychostimulants are associated with an increase in glutamate release in the ventral midbrain^44–46^. Consistent with these findings, the present study showed that increases in locomotor and rearing activities following challenge administration of nicotine were significantly correlated with an increase in glutamate concentration in the dorsal striatum. These findings suggest that an increased concentration of glutamate in the dorsal striatum causes an elevation of locomotor and rearing activities after challenge exposure to nicotine. Drug-associated cues increase glutamate release in the core of the NAc, which may be associated with relapse to nicotine-seeking behavior^23^. Blockade of glutamatergic neurotransmission in the NAc and VTA has been found to attenuate the reinstatement of drug-seeking behavior^47,48^. Similarly, in this study repeated and challenge administrations of nicotine after nicotine abstinence increased locomotor and rearing activities; increases that were paralleled by an increase in glutamate levels in the dorsal striatum. Taken together, these findings suggest that re-exposure to nicotine potentiates sensitivity to the glutamate response and causes behavioral changes in locomotor and rearing activities by recovering the glutamatergic and cholinergic systems, which are suppressed during nicotine withdrawal.

Cotinine is a major peripheral oxidative metabolite of nicotine in several animal species including rat^30,49^. Cotinine can be formed from nicotine in the brain or the periphery, and peripherally formed cotinine, can be redistributed to the brain through the blood-brain barrier^49–51^. A previous study demonstrated that systemic cotinine administration has no effect on locomotor activity in rat^52^. Consistent with these findings, the present data demonstrated that repeated cotinine administration did not induce alterations in locomotor and rearing activity levels. Taken together, these findings suggest that only nicotine, not nicotine metabolites delivered from periphery, stimulates nAChRs in the dorsal striatum. Such stimulation predominantly contributes to an increase in locomotor and rearing activities after repeated and challenge administrations of nicotine.

Previous studies have demonstrated that stimulation of nAChRs is involved in the hypersensitization of glutamate release^8,35,36^ and alteration of locomotion^53,54^ after repeated exposure to nicotine. For example, systemic administration of the nAChR antagonist, mecamylamine prior to nicotine administration attenuates nicotine-induced hyperactivity of locomotion in rat^53^. In addition, intra-VTA infusion of MLA attenuates the reinforcing effects of nicotine on brain reward in rat^55^. Consistent with these findings, the present data demonstrate that MLA pretreatment via peripheral or local routes can attenuate the nicotine challenge-induced increases in glutamate release in the dorsal striatum and locomotor and rearing activities. These findings suggest that challenge exposure to nicotine seems to induce behavioral hyperactivity via an α7 nAChR-linked glutamate response in the rat dorsal striatum. Collectively, these findings suggest that an increase in the glutamate response via stimulation of α7 nAChRs following nicotine challenge is a neurochemical event in the rat dorsal striatum, contributing to behavioral changes in locomotor and rearing activities.

## Methods

### Animals

Adult male Sprague-Dawley rats (200–230 g) were obtained from Hyo-Chang Science Co. (Daegu, South Korea). A total of 114 rats were used for six separate experiments in this study (Experiment 1, n = 20; Experiment 2, n = 30; Experiment 3, n = 12; Experiment 4, n = 8; Experiment 5, n = 20; Experiment 6, n = 24). All procedures involving animals were approved by the Institutional Animal Care and Use Committee and carried out in accordance with the provisions of the National Institutes of Health Guide for the Care and Use of Laboratory Animals. Experimental details related to handling of rats are described in Supplementary Methods.

### Drugs

Nicotine hydrogen tartrate salt, cotinine, and _L_-glutamic acid were purchased from Sigma-Aldrich (St. Louis, MO, USA). The potent α7 nAChRs antagonist, MLA, was purchased from Tocris Bioscience (Bristol, UK). The _L_-ascorbic acid was purchased from Duchefa Biochemie B.V. (Haarlem, Netherlands). Experimental details for the preparation of drugs are described in Supplementary Methods.

### Experimental designs

Six separate experiments were conducted to test the hypothesis that hyperactivation of the glutamate response linked to α7 nAChRs in the dorsal striatum is necessary for the nicotine challenge-induced locomotor and rearing activities. The first experiment was conducted to determine whether acute or repeated nicotine exposure alters the concentration of extracellular glutamate in freely moving rats. The second experiment was performed to determine whether nicotine withdrawal after repeated nicotine exposure or re-exposure to nicotine followed by nicotine withdrawal can influence glutamate concentration in freely moving rats. The third experiment was performed to determine whether repeated and challenge administrations of nicotine alter locomotor and rearing activity levels. The fourth experiment was performed to determine whether cotinine, a nicotine metabolite, administration after repeated nicotine exposure contributes to those behavioral changes. The fifth experiment was conducted to determine whether stimulation of α7 nAChRs contributes to the nicotine challenge-induced hyperactivation of the glutamate response in the dorsal striatum of freely moving rats. The final experiment was performed to determine whether α7 nAChR-mediated hyperactivity of the glutamate response in the dorsal striatum contributes to the nicotine challenge-induced behavioral changes. There were no injections of saline or nicotine during the withdrawal periods throughout the experiments.

Additional experimental details related to each experimental design are described in Supplementary Methods.

### Surgery for glutamate biosensing and drug infusion

For insertion of the glutamate biosensor, a BASi Rat Guide Cannula (Pinnacle Technology, Lawrence, KS, USA) was surgically implanted into the center of the right dorsal striatum (1.0 mm anterior to the bregma, 2.5 mm right of the midline, and 5 mm below the surface of the skull). The possibility of gliosis caused by the implantation of the guide cannula and insertion of the glutamate biosensor was verified by Nissl staining (Supplementary Fig. S3). The attached accessories for biosensing did not interrupt nicotine-dependent behaviors (Supplementary Fig. S1). A different set of experiment was performed for intracaudate infusion of MLA, a 22-gauge stainless steel infusion guide cannula (PlasticsOne, VA, USA) was implanted at the right dorsal striatum. Throughout the experiments, vehicle or MLA were infused unilaterally into the central part of the right dorsal striatum 5 min prior to the final administration of saline or nicotine with a volume of 1 μL at a rate of 0.5 μL/min in freely moving rats. Experimental details related to surgical procedures for glutamate biosensing and MLA infusion are described in Supplementary Methods.

### *In vitro* calibration and real-time glutamate biosensing

This study used commercial _L_-glutamate oxidase-based glutamate biosensors (glutamate biosensors) (Part #7002, Pinnacle Technology) and _L_-glutamate oxidase-free glutamate biosensors (glutamate null biosensors) (Pinnacle Technology). In brief, the platinum-iridium (Pt-Ir) wire (diameter: 0.18 mm) with a 1.0 mm sensing tip and the Ag/AgCl reference electrode were incorporated with the active electrode. On the active surface of the glutamate biosensors, glutamate oxidase converts the glutamate to α-ketoglutarate and H_2_O_2_, diffusing through the selective membrane to the Pt-Ir surface, where it is detected as an amperometric oxidation current generated by a +0.6 V applied potential^56^. Before and after calibrations were performed in PBS (pH 7.4) by gradually increasing the glutamate concentrations from 0 to 1, 2, 3, and 4 μM followed by a single addition of ascorbate (250 μM). The biological interference compound, ascorbate, did not interfere with glutamate detection, which is consistent with the results from other studies using the biosensor^20,56^. All analytical solutions were freshly prepared prior to before and after calibrations. Since the response sensitivities of the biosensor to glutamate depend directly on temperature^57^, all calibrations were performed at 37 °C with sufficient time to allow for the biosensor to reach a stable baseline. In this study, acute and repeated nicotine administration did not alter temperature in the dorsal striatum in freely moving rats (Supplementary Fig. S4). In addition, since glutamate biosensors showed a downward tendency to the current changes *in vivo*, rats were habituated in the home cage until decreasing current changes had relatively stabilized into the baseline for a minimum of 120 min after inserting the biosensors. When changes in the currents reached a relatively stable condition, real-time glutamate biosensing in the dorsal striatum of freely moving rats was conducted for 20 min following saline or nicotine administration in home cage. Since the baseline current of individual rats was slightly influenced by each glutamate biosensor, the absolute values of the current caused by saline or nicotine administration were transformed into the relative values of the current by normalizing the basal value to be 0 nA. These changes in the current of the dorsal striatum were then converted into changes in the concentrations of glutamate based on the sensitivity of each glutamate biosensor adjusted by its calibration plots. Data were sampled at 1 Hz with the SIRENIA acquisition software (version 1.6.1, Pinnacle Technology).

### Behavioral assessments

Under illuminated and sound-attenuated conditions, locomotor activity (total distance traveled by horizontal beam breaks in a consecutive order), and rearing activity (counts by vertical beam breaks) for stereotypy movement were evaluated after drug administration or withdrawal treatment. Activities were recorded in an open-field condition by using an infrared photocell-based, automated Opto-Varimex 4 Auto Track (Columbus Instruments, Columbus, OH, USA). Locomotor and rearing activities were recorded in 1 min intervals for 30 or 20 min before and after administration of saline or nicotine, respectively. The obtained data were then transferred from all sensors to a computer by using Opto-Varimex 4 Auto Track Rapid Release software (version 4.99B, Columbus Instruments). Additional experimental information for the procedures related to behavioral measurements are described in Supplementary Methods.

### Statistics

Statistical analysis was performed by using two-tailed unpaired *t* tests or one- or two-way ANOVA with repeated measures (RM) followed by Tukey’s or Bonferroni’s post hoc tests, respectively. Analysis was conducted using GraphPad Prism 6 (GraphPad Software, La Jolla, CA, USA). The data were expressed as the means ± SEM for each group (n = 4–6 per group). The level of statistical significance was set at p < 0.05. Experimental details related to the statistical analyses are described in Supplementary Methods.

## Electronic supplementary material

Supplementary Information
