# Supplementary material for: Behavioral changes after nicotine challenge are associated with α7 nicotinic acetylcholine receptor-stimulated glutamate release in the rat dorsal striatum
Source: Sci Rep. 2017 Nov 8;7:15009. doi: 10.1038/s41598-017-15161-7 (PMC5678080; doi:10.1038/s41598-017-15161-7)
Supplement: Supplementary file 1 — Supplementary Information [file 41598_2017_15161_MOESM1_ESM.doc]

**Supplementary Information**

**Behavioral changes after nicotine challenge are associated with α7 nicotinic acetylcholine receptor-stimulated glutamate release in the rat dorsal striatum**

**In Soo Ryu^1,5^, Jieun Kim^1^, Su Yeon Seo^1,6^, Ju Hwan Yang^1^, Jeong Hwan Oh^2^, Dong Kun Lee^3^, Hyun-Wook Cho^4^, Seong Shoon Yoon^5^, Joung-Wook Seo^5^, Suchan Chang^7^, Hee Young Kim^7^, Insop Shim^8^, Eun Sang Choe^1^,***

**Methods**

**Animals.** Adult male Sprague-Dawley rats initially weighing approximately 200-230 g (6 weeks) were purchased from Hyo-Chang Science Co. (Daegu, South Korea). Rats were separated into pairs and allowed to stay in a home cage for a minimum of 5 days to avoid environmental influences, such as stress. Food and water were available ad libitum. Rats were maintained on a regular light-dark cycle throughout the experimental period. In addition, temperature and humidity were maintained at 21-23°C and 45-55%, respectively. All animal experiments were conducted in a quiet room to minimize stress to the animals. All procedures involving animals were approved by the Institutional Animal Care and Use Committee and carried out in accordance with the provisions of the National Institutes of Health Guide for the Care and Use of Laboratory Animals.

**Drugs.** Nicotine hydrogen tartrate salt and cotinine were purchased from Sigma-Aldrich (St. Louis, MO, USA) dissolved in sterile 0.9% physiological saline, and adjusted to pH 7.2-7.4 with sodium hydroxide (NaOH). The doses of nicotine (0.4 mg/kg/day) and cotinine (10 mg/kg/day) for subcutaneous (s.c.) administration (1 μL/kg) were determined from previous studies^1-3^. The potent α7 nAChRs antagonist, methyllycaconitine citrate (MLA), was purchased from Tocris Bioscience (Bristol, UK). MLA was dissolved in the artificial cerebro-spinal fluid (aCSF) containing (mM) 123 NaCl, 0.86 CaCl_2_, 3.0 KCl, 0.89 MgCl_2_, 0.50 NaH_2_PO_4_, and 0.25 Na_2_PO_4_ aerated with 95% O_2_/5% CO_2_, pH 7.2 to 7.4 or NaCl. The aCSF solution was conducted as the vehicle control for the given MLA. The doses of MLA for intraperitoneal (i.p.) administration (10 mg/kg/day) and unilateral intracaudate infusion (10 μg/μL/side) were determined from previous studies^4,5^. The _L_-glutamic acid (Sigma-Aldrich) and _L_-ascorbic acid (Duchefa Biochemie B.V., Haarlem, Netherlands) were dissolved in phosphate buffered saline (PBS, pH 7.4) to make the standard glutamate solution and interfering analytical solution, respectively, for *in vitro* calibration of glutamate biosensors. All working solutions of drugs were always prepared fresh immediately just prior to every experiment.

**Experimental design.** Six separate experiments were conducted to test the hypothesis that the hyperactivation of glutamate response linked to α7 nAChRs in the dorsal striatum is necessary for the reinstatement of nicotine-induced sensitization of locomotor and rearing activities. The first experiment was conducted to determine whether acute or repeated nicotine exposure alters the concentrations of extracellular glutamate. We performed real-time measures of glutamate biosensing for 20 min after acute and repeated saline or nicotine administrations in freely moving rats. Rats were randomly divided into four different groups: 1) acute saline group (s.c.); 2) acute nicotine group (s.c.); 3) 14 days repeated saline group (s.c.); and 4) 14 days repeated nicotine group (0.4 mg/kg/day, s.c.) (n=5 per group).

The second experiment was performed to determine whether nicotine withdrawal after repeated nicotine exposure or re-exposure to nicotine followed by nicotine withdrawal influences the concentrations of glutamate in freely moving rats. We performed real-time measures of glutamate biosensing for 20 min after the 1^st^ day of withdrawal, the 6^th^ day of withdrawal, or challenge administration of saline or nicotine. Rats were divided into six different groups: 1) 14 days repeated saline (s.c.) + 1^st^ day of withdrawal group; 2) 14 days repeated nicotine (0.4 mg/kg/day, s.c.) + 1^st^ day of withdrawal group; 3) 14 days repeated saline (s.c.) + 6^th^ day of withdrawal group; 4) 14 days repeated nicotine (0.4 mg/kg/day, s.c.) + 6^th^ day of withdrawal group; 5) 14 days repeated saline (s.c.) + 6^th^ day of withdrawal + saline challenge group (s.c.) (n = 5); and 6) 14 days repeated nicotine (0.4 mg/kg/day, s.c.) + 6^th^ day of withdrawal + nicotine challenge group (0.4 mg/kg/day, s.c.) (n=5 per group).

The third experiment was performed to determine whether the repeated and challenge administrations of nicotine alters sensitization of locomotor and rearing activity. We measured the changes of locomotor and rearing activities for 20 min after every administration or withdrawal treatment of saline or nicotine for 21 days. Rats were divided into two different groups: 1) 14 days repeated saline (s.c.) + 6^th^ day of withdrawal + saline challenge group (s.c.); and 2) 14 days repeated nicotine (0.4 mg/kg/day, s.c.) + 6^th^ day of withdrawal + nicotine challenge group (0.4 mg/kg/day, s.c.) (n=6 per group).

The fourth experiment was performed to determine whether cotinine, a nicotine metabolite, after repeated nicotine exposure contributes to behavioral sensitization. We measured the changes of locomotor and rearing activities for 20 min after the final injection of saline or cotinine on the 14^th^ day. Rats were divided into two different groups: 1) 14 days repeated saline group (s.c.); and 2) 14 days repeated cotinine group (10 mg/kg/day, s.c.) (n=4 per group).

The fifth experiment was conducted to determine whether stimulation of α7 nAChRs contributes to the nicotine challenge-induced hyperactivation of glutamate response in the dorsal striatum of freely moving rats. We performed real-time measures of glutamate biosensing 20 min after nicotine challenge administration, followed by systemic administration of the α7 nAChR antagonist, MLA, or vehicle. Rats were divided into four different groups: 1) 14 days repeated nicotine (0.4 mg/kg/day, s.c.) + 6^th^ day of withdrawal + vehicle pretreatment (i.p.) + saline challenge (s.c.); 2) 14 days repeated nicotine (0.4 mg/kg/day, s.c.) + 6^th^ day of withdrawal + MLA pretreatment (10 mg/kg/day, i.p.) + saline challenge (s.c.); 3) 14 days repeated nicotine (0.4 mg/kg/day, s.c.) + 6^th^ day of withdrawal + vehicle pretreatment (i.p.) + nicotine challenge (0.4 mg/kg/day, s.c.); and 4) 14 days repeated nicotine (0.4 mg/kg/day, s.c.) + 6^th^ day of withdrawal + MLA pretreatment (10 mg/kg/day, i.p.) + nicotine challenge (0.4 mg/kg/day, s.c.) (n=5 per group).

The final experiment was performed to determine whether α7 nAChR-mediated hyperactivity of glutamate response in the dorsal striatum contributes to the nicotine challenge-induced behavioral sensitization. We measured the changes of locomotor and rearing activities for 20 min after challenge administration of saline or nicotine followed by intracaudate infusion of MLA, or vehicle. Rats were divided into four different groups: 1) 14 days repeated nicotine (0.4 mg/kg/day, s.c.) + 6^th^ day of withdrawal + vehicle pretreatment (intracaudate route) + saline challenge (s.c.); 2) 14 days repeated nicotine (0.4 mg/kg/day, s.c.) + 6^th^ day of withdrawal + MLA pretreatment (10 μg/μL, intracaudate route) + saline challenge (s.c.); 3) 14 days repeated nicotine (0.4 mg/kg/day, s.c.) + 6^th^ day of withdrawal + vehicle pretreatment (intracaudate route) + nicotine challenge (0.4 mg/kg/day, s.c.); and 4) 14 days repeated nicotine (0.4 mg/kg/day, s.c.) + 6^th^ day of withdrawal + MLA pretreatment (10 μg/μL, intracaudate route) + nicotine challenge (0.4 mg/kg/day, s.c.) (n=6 per group).

**Surgery for glutamate biosensing and drug infusion.** Rats were anesthetized with a mixture of Zoletil 50 (18.75 mg/kg) (Virbac Korea, Seoul, South Korea) and Rompun (5.8 mg/kg) (Bayer Korea, Seoul, South Korea) via i.p. injections. Rats were then placed in a stereotaxic apparatus. Under aseptic conditions, a BASi Rat Guide Cannula (Part #7030, Pinnacle Technology, Lawrence, KS, USA) (inner diameter: 0.7 mm, 10 mm in length) was surgically implanted into the center of the right dorsal striatum (1.0 mm anterior to the bregma, 2.5 mm right of the midline, and 5 mm below the surface of the skull) for insertion of the glutamate biosensor. BLE Rat Hat Bottoms (Part #8108, Pinnacle Technology) were positioned on the hat of the rat to enable the placement of potentiostat (Part #8172, No. 9225/9226, Pinnacle Technology), after which the BLE Rat Hat Bottoms were covered with the BLE Rat Hat Tops (Part #8107, Pinnacle Technology) until glutamate biosensing. After surgery, rats were given a minimum of 5 days of postoperative recovery in the cage, and then treated with 0.1 ml of gentamycin (i.p.) (Eagle Vet, Seoul, South Korea) prior to the first saline or nicotine administration. The physical accuracy of glutamate biosensing was verified by the reconstruction of the guide cannula placements (Supplementary Fig. S3). The possibility of gliosis caused by the implantation of the guide cannula and insertion of the glutamate biosensor was verified by Nissl staining (Supplementary Fig. S3). For intracaudate infusion of the drugs, a 22-gauge stainless steel infusion guide cannula (Part #C313G/SPC, PlasticsOne, VA, USA) (inner diameter: 0.39 mm, 5 mm in length) was implanted at the right dorsal striatum (coordinates were same as above). The guide cannula was sealed with a 28-gauge stainless steel infusion dummy cannula (Part #C323DC/SPC, PlasticsOne) (inner diameter: 0.36 mm, 5 mm in length). Other processes were the same as the surgery for glutamate biosensing.

**Measurement of temperature in the dorsal striatum.** Under the anesthesia, guide cannula (custom-made) was surgically implanted into the center of the right dorsal striatum (1.0 mm anterior to the bregma, 2.5 mm right of the midline, and 5 mm below the surface of the skull) for insertion of the thermocouple needle microprobe (NJ-07013, WPI, Sarasota, USA). Other surgical processes were the same as the surgery for glutamate biosensing. In order to measure temperature in the dorsal striatum in freely moving rats, the thermocouple needle microprobe was slowly inserted into the dorsal striatum. Then the thermocouple probe was connected to a BAT-12 digital thermometer (Physitemp, New Jersey, USA) and a data acquisition system (Powerlab, ADinstruments, Australia). Changes in temperature in the dorsal striatum were recorded for 20 min after acute and repeated nicotine administration.

**Intracaudate infusion of MLA.** Intracaudate infusion of MLA was routinely performed as previously described^6^. On the day of the experiment, the infusion dummy cannula was replaced with a 28-gauge stainless steel infusion internal cannula (Part #C313I/SPC, PasticsOne) (inner diameter: 0.36 mm, 5.5 mm in length). The progress of injection was monitored by observing the movement of a small air bubble through a length of the precalibrated PE-10 tubing inserted between the infusion internal cannula and a 2.5 μL Hamilton microsyringe (Hamilton, NV, USA). After completing the injection, the injector was left in place for an additional 5 min to prevent any possible backflow of the solution along with the injection tract.

**Behavioral assessments.** Behavioral assessments were performed as previously described^6^. Under the illuminated and sound-attenuated conditions, the locomotor activity (total distance travelled by horizontal beam breaks in a consecutive order) and rearing activity for stereotypy movement (counts by vertical beam breaks) were evaluated in an open-field with an infrared photocell-based, automated Opto-Varimex 4 Auto Track (Columbus Instruments, Columbus, OH, USA) after drug administration or withdrawal treatment. Rats were acclimated in a locomotion test chamber (44.5 cm x 44.5 cm) for a minimum of 6 days to avoid environmental variations prior to the first saline or nicotine administration. Three pairs of sensors were positioned on the x-, y- (horizontal), and z- (vertical, placed above the animal’s normal height) axes to provide coordinates of the travelled distance and rearing of animals. Each sensor produces 16 infrared light beams that intersect at the animal cage (beam scan rate = 10 Hz), and the Auto-Track system senses the presence of animals by receiving the infrared beam interruptions. The locomotor and rearing activities were recorded in 1 min intervals for 30 and 20 min before and after administration of saline or nicotine, respectively, which were then transferred from all sensors to a computer with Opto-Varimex 4 Auto Track Rapid Release software (version 4.99B, Columbus Instruments).

**Statistics.** For statistical analysis, the detected currents by the glutamate biosensors were converted into glutamate concentrations. Alterations in the locomotor and rearing activities were recorded as the total travelled distance (cm) and rearing (count) in 5 min intervals for 40 min. Statistical significance between the groups was determined by two-tailed unpaired t test, and one- or two-way ANOVA with repeated measures (RM), followed by Tukey’s or Bonferroni’s post hoc test, respectively. Analysis was conducted using GraphPad Prism 6 (GraphPad Software, La Jolla, CA, USA). The data were expressed as the means ± SEM for each group (n=4-6 per group). The level of statistical significance was set at p<0.05.

**References**

1. Matta, S. G. *et al*. Guidelines on nicotine dose selection for *in vivo* research. *Psychopharmacology (Berl)* **190**, 269-319 (2007).

2. Harris, A. C. *et al*. Effects of nicotine and minor tobacco alkaloids on intracranial-self-stimulation in rats. *Drug Alcohol Depend* **153**, 330-334 (2015).

3. Wiley, J. L., Marusich, J. A., Thomas, B. F. & Jackson, K. J. Determination of behaviorally effective tobacco constituent doses in rats. *Nicotine Tob Res* **17**, 368-371 (2015).

4. Panagis, G., Kastellakis, A., Spyraki, C. & Nomikos, G. Effects of methyllycaconitine (MLA), an alpha 7 nicotinic receptor antagonist, on nicotine- and cocaine-induced potentiation of brain stimulation reward. *Psychopharmacology (Berl)* **149**, 388-396 (2000).

5. Boccia, M. M., Blake, M. G., Krawczyk, M. C. & Baratti, C. M. Hippocampal α7 nicotinic receptors modulate memory reconsolidation of an inhibitory avoidance task in mice. *Neuroscience* **171**, 531-543 (2010).

6. Oh, J. H. *et al*. Dopamine D4 receptors linked to protein kinase G are required for changes in dopamine release followed by locomotor activity after repeated cocaine administration. *Exp Brain Res* **233**, 1511-1518 (2015).

**Supplementary Figure S1.** Changes in locomotor and rearing activities after repeated nicotine administration in non-operated (non-OP) and operated (OP) groups. Repeated nicotine administration significantly increased in locomotor (F_(2, 19)_=46.04, p<0.0001) (a) and rearing (F_(2, 19)_=21.23, p<0.0001) (c) activities in non-OP and OP rats as compared with the control groups. Similar results were obtained from 5 min interval analysis: locomotor activity (P1, F_(2, 19)_=32.23, p<0.0001; P2, F_(2, 19)_=22.85, p<0.0001; P3, F_(2, 19)_=19.01, p<0.001; P4, F_(2, 19)_=12.76, p=0.0003) (b); rearing activity (P1, F_(2, 19)_=5.634, p=0.0120; P2, F_(2, 19)_=20.07, p<0.0001; P3, F_(2, 19)_=21.63, p<0.001; P4, F_(2, 19)_=7.217, p=0.0047) (d). There were no changes in locomotor and rearing activities between non-OP and OP groups throughout all periods (b, d). All values are represented as the mean ± SEM. *p<0.05 versus 14 days repeated saline control group. P1, 0-5 min; P2, 5-10 min; P3, 10-15 min; P4, 15-20 min; n=6-10 per group.

**Supplementary Figure S2.** Changes in the locomotor and rearing activities after 14 days repeated cotinine administration. Timeline for behavioral assessments following 14 days repeated saline or cotinine (10 mg/kg/day) administration (a). Changes in the locomotor activity (b, c) and rearing activity (d, e) for 40 min following 14 days repeated saline or cotinine administration. P1, 0-5 min; P2, 5-10 min; P3, 10-15 min; P4, 15-20 min. n=4 per group.

**Supplementary Figure S3.** The placements of glutamate biosensors in the dorsal striatum. A Nissl-stained brain section showing statistics of the placements of glutamate biosensors (black arrows) implanted unilaterally into the center of the right dorsal striatum (CPu) and verification of gliosis caused by implantation of the biosensor. cc, corpus callosum; NAc, nucleus accumbens.


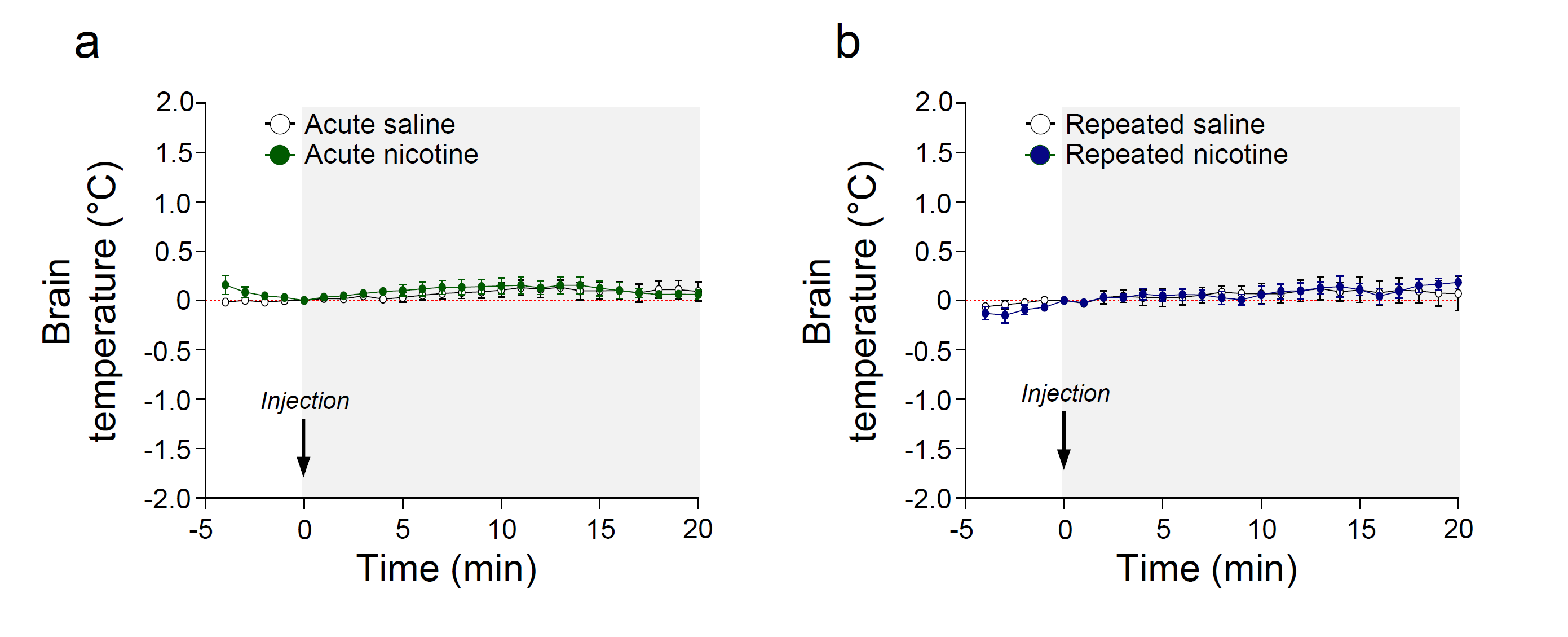


**Supplementary Figure S4.** Changes in temperature in the dorsal striatum after acute (a) and repeated (b) nicotine (0.4 mg/kg/day) administration in freely moving rats. All values are represented as the mean ± SEM. n=4-5 per group.

**Supplementary Table S1.** Rate of changes in the glutamate concentrations in the dorsal striatum obtained from real-time glutamate biosensing at 5 min intervals up to 20 min after saline or nicotine administration

| Group | Glutamate concentrations (nM) | | | |
| --- | --- | --- | --- | --- |
|  | 5 min | 10 min | 15 min | 20 min |
| Acute saline | -6.30 ± 18.66 | 12.89 ± 29.76 | 23.21 ± 22.22 | -12.90 ± 15.19 |
| Acute nicotine | -25.96 ± 12.58 | -174.70 ± 10.19^*^ | -45.89 ± 26.19 | -24.65 ± 21.11 |
| 14 days repeated saline | -15.85 ± 22.82 | -19.20 ± 21.93 | -5.62 ± 15.54 | -25.95 ± 15.54 |
| 14 days repeated nicotine | 88.97 ± 16.31^*^ | 60.57 ± 16.29^*^ | 51.71 ± 25.89 | 12.33 ± 8.63 |
| 14 days repeated saline +  1^st^ day of withdrawal | -207.00 ± 93.30 | -190.6 ± 49.44 | -145.70 ± 56.46 | -85.72 ± 26.70 |
| 14 days repeated nicotine +  1^st^ day of withdrawal | -212.20 ± 58.67 | -263.6 ± 62.87 | -118.70 ± 60.05 | -26.11 ± 10.25 |
| 14 days repeated saline +  6^th^ day of withdrawal | -182.90 ± 56.73 | -208.90 ± 58.71 | -238.70 ± 79.55 | -79.55 ± 27.41 |
| 14 days repeated nicotine +  6^th^ day of withdrawal | -162.80 ± 26.22 | -210.00 ± 14.72 | -223.40 ± 9.70 | -121.30 ± 59.17 |
| 14 days repeated saline + 6^th^ day of withdrawal + challenge saline | -12.28 ± 22.56 | -28.73 ± 18.73 | -1.58 ± 21.17 | -24.02 ± 14.96 |
| 14 days repeated nicotine +  6^th^ day of withdrawal + challenge nicotine | 119.10 ± 42.24^*^ | 34.34 ± 26.74 | 40.41 ± 23.33 | -24.41 ± 33.83 |
| 14 days repeated saline  (glutamate null biosensors) | -13.80 ± 4.95 | -19.72 ± 8.12 | -25.76 ± 7.14 | -20.77 ± 6.53 |
| 14 days repeated nicotine  (glutamate null biosensors) | -5.02 ± 8.09 | -16.12 ± 9.47 | -23.83 ± 7.95 | -21.15 ± 5.86 |

* represents a significant increase in the rate of changes in extracellular glutamate concentrations as compared with the control groups.

**Supplementary Table S2.** Changes in the locomotor and rearing activities at 5 min intervals up to 20 min after saline or nicotine administration

| Group | Locomotor activity (cm) | | | |
| --- | --- | --- | --- | --- |
|  | 5 min | 10 min | 15 min | 20 min |
| Acute saline | 406.20 ± 89.82 | 366.50 ± 109.90 | 174.30 ± 49.90 | 18.50 ± 6.45 |
| Acute nicotine | 370.20 ± 76.53 | 251.30 ± 62.98 | 235.30 ± 49.71 | 97.67 ± 50.76^*^ |
| 14 days repeated saline | 275.30 ± 52.00 | 81.33 ± 30.56 | 94.33 ± 50.93 | 99.33 ± 70.32 |
| 14 days repeated nicotine | 1504.00 ± 187.50^*^ | 1105.00 ± 164.50^*^ | 847.00 ± 150.80^*^ | 384.50 ± 101.50^*^ |
| 14 days repeated saline +  1^st^ day of withdrawal | 549.70 ± 158.70 | 178.70 ± 68.46 | 48.17 ± 20.71 | 94.60 ± 88.15 |
| 14 days repeated nicotine +  1^st^ day of withdrawal | 608.50 ± 221.60 | 216.70 ± 105.50 | 77.50 ± 63.77 | 22.17 ± 18.04 |
| 14 days repeated saline +  6^th^ day of withdrawal | 250.50 ± 42.99 | 16.50 ± 9.06 | 108.0 ± 76.47 | 3.00 ± 1.09 |
| 14 days repeated nicotine +  6^th^ day of withdrawal | 311.70 ± 57.28 | 9.83 ± 3.06 | 5.16 ± 2.19 | 2.33 ± 1.28 |
| 14 days repeated saline +  6^th^ day of withdrawal + challenge saline | 275.30 ± 52.00 | 81.33 ± 30.56 | 94.33 ± 50.93 | 99.33 ± 70.32 |
| 14 days repeated nicotine +  6^th^ day of withdrawal + challenge nicotine | 1481.00 ± 147.50^*^ | 934.80 ± 166.70^*^ | 684.30 ±97.32^*^ | 423.50 ±129.30 |
| Group | Rearing activity (count) | | | |
|  | 5 min | 10 min | 15 min | 20 min |
| Acute saline | 88.33 ± 23.17 | 85.83 ± 15.72 | 35.00 ± 12.32 | 0.66 ± 0.67 |
| Acute nicotine | 52.33 ± 19.01 | 16.67 ± 9.28 | 24.17 ±16.03 | 9.83 ± 4.73 |
| 14 days repeated saline | 161.00 ± 44.14 | 26.67 ± 13.59 | 2.50 ± 2.50 | 20.67 ± 17.34 |
| 14 days repeated nicotine | 337.80 ± 41.77^*^ | 267.8 ± 42.98^*^ | 234.00 ± 44.50^*^ | 112.50 ± 24.80^*^ |
| 14 days repeated saline +  1^st^ day of withdrawal | 133.50 ± 33.02 | 46.83 ± 16.51 | 11.17 ± 6.24 | 11.83 ± 10.50 |
| 14 days repeated nicotine +  1^st^ day of withdrawal | 70.83 ± 18.08 | 9.00 ± 9.00 | 11.50 ± 11.50 | 3.50 ± 2.54 |
| 14 days repeated saline +  6^th^ day of withdrawal | 46.17 ± 10.47 | 7.16 ± 6.57 | 6.33 ± 4.29 | 1.83 ± 0.89 |
| 14 days repeated nicotine +  6^th^ day of withdrawal | 50.17 ± 19.91 | 1.16 ± 0.98 | 1.17 ± 1.17 | 1.21 ± 0.21 |
| 14 days repeated saline +  6^th^ day of withdrawal + challenge saline | 183.00 ± 48.78 | 40.67 ± 9.79 | 39.00 ± 18.16 | 39.67 ± 21.95 |
| 14 days repeated nicotine +  6^th^ day of withdrawal + challenge nicotine | 535.20 ± 101.80^*^ | 302.80 ± 60.95^*^ | 214.50 ±27.62^*^ | 152.20 ± 24.10^*^ |

* represents a significant increase in the locomotor and rearing activities as compared with the control groups.

**Supplementary Table S3.** Changes in the locomotor and rearing activities at 5 min intervals up to 20 min after 14 days repeated saline or cotinine administration

| Group | Locomotor activity (cm) | | | |
| --- | --- | --- | --- | --- |
|  | 5 min | 10 min | 15 min | 20 min |
| 14 days repeated saline | 303.00 ± 88.15 | 14.25 ± 8.44 | 4.75 ± 2.83 | 8.00 ± 2.85 |
| 14 days repeated cotinine | 310.00 ± 108.10 | 8.75 ± 6.62 | 8.00 ± 6.04 | 10.75 ± 4.99 |
| Group | Rearing activity (count) | | | |
|  | 5 min | 10 min | 15 min | 20 min |
| 14 days repeated saline | 79.50 ± 10.88 | 5.00 ± 4.00 | 1.02 ± 0.08 | 1.00 ± 0.57 |
| 14 days repeated cotinine | 65.25 ± 20.81 | 13.75 ± 3.25 | 1.75 ± 0.75 | 1.50 ± 0.86 |

**Supplementary Table S4.** Rate of changes in the glutamate concentrations in the dorsal striatum obtained from real-time glutamate biosensing at a 5 min intervals up to 20 min after saline or nicotine challenge administration followed by pretreatment with vehicle or MLA

| Group | Glutamate concentrations (nM) | | | |
| --- | --- | --- | --- | --- |
|  | 5 min | 10 min | 15 min | 20 min |
| 14 days repeated nicotine +  6^th^ day of withdrawal + vehicle + challenge saline | -79.45 ± 4.02 | -31.02 ± 34.81 | -25.59 ± 30.20 | -62.45 ± 39.42 |
| 14 days repeated nicotine +  6^th^ day of withdrawal + MLA + challenge saline | -24.65 ± 46.95 | -8.80 ± 48.05 | 9.22 ± 28.83 | -26.70 ± 26.82 |
| 14 days repeated nicotine +  6^th^ day of withdrawal + vehicle + challenge nicotine | 159.40 ± 39.50^*^ | 96.02 ± 53.01 | -16.00 ± 56.18 | -25.351 ± 27.60 |
| 14 days repeated nicotine +  6^th^ day of withdrawal + MLA + challenge nicotine | 57.72 ± 11.30^#^ | -54.19 ± 23.05^#^ | -48.76 ± 5.46 | -44.36 ± 20.8 |

*, ^#^ represent a significant increase or decrease in the rate of changes in extracellular glutamate concentrations as compared with the control groups.

**Supplementary Table S5.** Changes in the locomotor and rearing activities at 5 min intervals up to 20 min after saline or nicotine challenge administration followed by pretreatment with vehicle or MLA

| Group | Locomotor activity (cm) | | | |
| --- | --- | --- | --- | --- |
|  | 5 min | 10 min | 15 min | 20 min |
| 14 days repeated nicotine +  6^th^ day of withdrawal + vehicle + challenge saline | 443.20 ± 109.50 | 287.50 ± 72.45 | 235.70 ± 63.70 | 159.80 ± 65.50 |
| 14 days repeated nicotine +  6^th^ day of withdrawal + MLA +  challenge saline | 583.50 ± 71.40 | 466.00 ± 149.00 | 167.80 ± 97.07 | 70.83 ± 41.24 |
| 14 days repeated nicotine +  6^th^ day of withdrawal + vehicle + challenge nicotine | 1307.00 ± 124.00^*^ | 768.50 ± 38.98^*^ | 658.70 ± 53.39^*^ | 567.00 ± 49.58^*^ |
| 14 days repeated nicotine +  6^th^ day of withdrawal + MLA +  challenge nicotine | 794.00 ± 124.00^*#^ | 763.30 ± 133.70^*^ | 611.80 ± 86.59^*^ | 426.70 ± 50.46^*^ |
| Groups | Rearing activity (count) | | | |
|  | 5 min | 10 min | 15 min | 20 min |
| 14 days repeated nicotine +  6^th^ day of withdrawal + vehicle + challenge saline | 95.17 ± 25.92 | 56.00 ± 15.44 | 45.83 ± 12.30 | 39.67 ± 21.95 |
| 14 days repeated nicotine +  6^th^ day of withdrawal + MLA +  challenge saline | 99.50 ± 11.90 | 42.67 ± 9.37 | 40.67 ± 28.26 | 25.83 ± 15.51 |
| 14 days repeated nicotine +  6^th^ day of withdrawal + vehicle + challenge nicotine | 297.30 ± 28.81^*^ | 170.00 ± 27.11^*^ | 133.20 ± 24.53^*^ | 161.30 ± 38.07^*^ |
| 14 days repeated nicotine +  6^th^ day of withdrawal + MLA +  challenge nicotine | 81.67 ± 27.81^#^ | 94.17 ± 30.48^#^ | 46.33 ± 23.84^#*^ | 48.00 ± 24.90^#^ |

*, ^#^ represent a significant increase or a decrease in the locomotor and rearing activities as compared with the control groups.
